# Attacked ravens flexibly adjust signalling behaviour according to audience composition

**DOI:** 10.1098/rspb.2018.0375

**Published:** 2018-06-06

**Authors:** Georgine Szipl, Eva Ringler, Thomas Bugnyar

**Affiliations:** 1 Department of Cognitive Biology, University of Vienna, Althanstrasse 14, A-1090 Vienna, Austria; 2 Department of Integrative Zoology, University of Vienna, Althanstrasse 14, A-1090 Vienna, Austria; 3 Konrad Lorenz Research Station, Core Facility, University of Vienna, Fischerau 11, A-4645 Gruenau, Austria; 4 Messerli Research Institute, University of Veterinary Medicine Vienna, Medical University of Vienna, and University of Vienna, Veterinaerplatz 1, A-1210 Vienna, Austria

**Keywords:** audience, communication, triadic awareness, raven, *Corvus corax*

## Abstract

A fundamental attribute of social intelligence is the ability to monitor third-party relationships, which has been repeatedly demonstrated in primates, and recently also in captive ravens. It is yet unknown how ravens make use of this ability when dealing with different types of social relationships simultaneously during complex real-life situations. Free-ranging non-breeder ravens live in societies characterized by high fission–fusion dynamics and structured by age, pair-bond status and kinship. Here, we show that free-ranging ravens modify communication during conflicts according to audience composition. When being attacked by dominant conspecifics, victims of aggression signal their distress via defensive calls. Victims increased call rates when their kin were in the bystander audience, but reduced call rates when the bystanders were bonding partners of their aggressors. Hence, ravens use social knowledge flexibly and probably based on their own need (i.e. alert nearby allies and avoid alerting nearby rivals).

## 1. Introduction

Sociality is thought to have driven the evolution of cognitive abilities in primates [1–4] and other mammals (e.g. cetaceans [5], carnivores and ungulates [6]), and possibly also in birds like parrots and corvids [7] (but see [8,9] for an argument against this notion in hyenas). Intellectually demanding challenges, such as the formation of individualized social relationships (social bonds) and the competence of dealing with others and their bonds [10,11], probably require increased problem-solving skills. Cognitively demanding societies are characterized by large structured groups and/or high fission–fusion dynamics, with high spatio-temporal variation in group size and group composition [12]. In these societies, one of the major challenges is to keep track of own and others' social relationships (i.e. whether or not social allies are around that may provide support during social conflicts and resource competition) [12].

Communicative abilities tend to become more complex with increasing cognitive abilities both at a repertoire and a call-type level [13–16]. As animal communication is an interplay between several individuals in signalling and receiving range of each other [17], apparent bystanders can influence the behaviour of the interacting individuals, even though the bystanders may not be directly involved in an interaction. This widespread phenomenon can be found in various animals and contexts, and is commonly referred to as the audience effect [18]. For instance, the likelihood and intensity of alarm calling and food calling depends on the presence of group members [19] or potential mating partners [20] in chicken, and kin/offspring [21], dominant individuals [22] or important social partners [23,24] in primates. Audience effects can also be used to investigate an individuals' ability to recognize others' social relationships and monitor their social interactions [25]. There is evidence that primates and other highly social mammals, like dolphins and hyenas, recognize social relationships of others through eavesdropping on their communicative exchanges [26]. In songbirds, eavesdropping has been studied in the context of mate choice and territorial defence, revealing that individuals are able to gather information about the relative strength or quality of neighbouring competitors through eavesdropping [27–29], and even combine gathered information with their own direct experience with either of the competitors [30]. The ability to assess unknown relationships via transitive inference was demonstrated in several captive corvids [31–33]. Furthermore, playback studies on captive ravens revealed long-term memory for affiliates [34] and recognition of group members' dominance relations in simulated encounters [35]. Taken together, these studies suggest that corvids, much like socially complex mammals, have the capacity to recognize own and others' relationships. Unlike for mammals, however, hardly anything is known about corvids' ability to apply this knowledge under field conditions, when they are part of a highly dynamic fission–fusion society and have to deal with different types of social relationships simultaneously.

Common ravens (*Corvus corax*) live in long-term monogamous relationships with their bonding partners, and defend a large territory year-round when they become territorial breeders [36]. Until then, ravens aggregate in vagrant non-breeder groups with constantly changing group sizes and compositions over time [37,38]. Nonetheless, non-breeder groups are structured by age, pair-bond status and kinship [36,39–41]. The importance of strong social bonds with siblings and affiliates was demonstrated in studies on captive birds: during and after intense conflicts, ravens were shown to provide agonistic support [42] and bystander affiliation [43] to valuable social partners (i.e. kin and bonding partners). A study in free-ranging ravens showed that bonded birds are higher in rank than singletons, and thus more successful when competing over food [41].

Non-breeder ravens gather in large numbers at feeding sites, thereby fighting for access to food defended by territory holders or dominant conspecifics [44,45]. Attacked ravens may utter defensive calls, which raise the attention of bystanders and attract nearby conspecifics [35,46]. The number of calls uttered by victims increases with the level of aggression, and victims of aggression were more likely to receive social support by a third-party bystander when calling compared with when victims did not call [47].

In this study, we examined free-ranging ravens' ability to flexibly adjust their signalling behaviour based on their knowledge of others' social relationships when attacked. Specifically, we investigated variations in victims' call rates during conflicts of moderate intensity with respect to audience composition (i.e. whether the presence of kin or bonding partners of the victims and of the aggressors in the bystander audience would have an impact on victims' call rates). If audience composition affected victims' call rates, the attacked birds should decrease call rates when bonding partners or kin *of the aggressors* were in the audience, as those could provide support to the aggressors. On the other hand, when the audience contained bonding partners or kin *of the victims*, victims’ call rates should increase as to alert individuals that would support them. Many factors aside from audience composition could influence victims' signalling behaviour. In ravens, dominance rank is determined by sex, age and bonding status, with males dominating females, adults dominating subadults and juveniles, and bonded birds dominating non-bonded birds [41]. Furthermore, the strength of a social relationship may determine whether support is provided to either of the opponents. We expected relationships among kin to be highly valuable for any raven due to shared inclusive fitness [48]. The value of social bonds with non-kin, however, could vary for each of the bonding partners [49]. We therefore conducted focal observations on dyadic affiliative interactions throughout the study period to determine the value of social bonds. Aside from age, sex, rank and bonding status, victims' signalling behaviour could be altered in response to the size of the audience, or the behaviour of valuable social partners in close proximity. We therefore additionally investigated whether the total group size and the close proximity of victims' and aggressors’ kin and bonding partners affected the victims' signalling behaviour.

## 2. Material and methods

### (a) Data collection

The study was conducted from August 2010 to July 2011 at the Cumberland Gamepark in Gruenau im Almtal, Upper Austria. Free-ranging ravens gather at the enclosure of the wild boars during the morning feedings to scrounge food from them, and can be observed year-round as the birds are well-habituated to humans and experimental equipment. In the course of a long-term monitoring project of this population of ravens, 150 individuals were caught and marked with individual colour rings and a metal ring from the German bird ringing station (Vogelwarte Radolfzell). During this standardized marking procedure, 50–200 µl blood was taken from the alar vein for sexing and analysis of relatedness. Age class (juvenile, subadult and adult) was determined by the coloration of the inner beak, which turns from pink (juvenile) to black (adult) with increasing age [50]. An average ± s.e. of 21.5 ± 0.36 marked individuals was present per day throughout the study period.

Agonistic interactions in ravens can be categorized by the intensity of the attack. During fights, both the initiating individual (referred to as ‘aggressor') and the targeted individual (the ‘victim') apply contact aggression and make use of their beak and claws [51]. During forced retreats, the victim retreats after being threatened by the aggressor [51]. During approach–retreat interactions, the victim retreats from the aggressor immediately after its approach without any physical aggression [51]. As the intensity of the attack was shown to alter the acoustic structure of defensive calls [47], we focused on agonistic interactions of moderate intensity in this study, namely forced retreats.

Data were collected at the wild boars' enclosure using binoculars and a voice recorder. Feedings were additionally videotaped using a high-definition camcorder (Canon HF-11 HD; Canon Inc., Japan) to allow for detailed frame-by-frame analysis of the interactions, the opponents involved, and the bystanders. From these videos, we extracted 103 dyadic forced retreat interactions of 40 individuals in which the victims uttered defensive calling, and both aggressor and victim could be identified individually. Defensive calls are loud and conspicuous calls which may be uttered by victims as single calls or sequences of several calls when retreating from aggressors [52,53]. The calling individual could be identified undoubtedly because its beak was wide open. Aggressors do not utter defensive calls, and aside from defensive calls, ravens do not utter other calls at the feeding grounds while trying to get access to food. The response of recipients to defensive calls may vary from subtle changes in head and body orientation to active intervention into the conflict [47]. Unlike interventions, the subtle responses to calls are difficult to measure under crowd foraging conditions.

For each forced retreat, we noted the number of calls emitted and the duration of the encounter (starting when the approaching aggressor was in contact distance to the victim, and ending when the victim moved out of reach of the aggressor) to calculate call rates. Additional data collected were the total number of bystander birds present, defined as birds within a radius of approximately 25 m to the opponents, their identity, as well as the identity of bystander birds in close proximity (within two body's lengths, i.e. 1 m) to the opponents. Behavioural responses of potential recipients in the audience were not measured, as subtle behavioural changes could not be assessed reliably from the video tapes. Inter-observer reliability was conducted on a subset of the data used in this study (11 cases) and randomly selected agonistic interactions (five cases) by GS and a second observer. For the type of aggression and the number of calls emitted, 100% agreement was achieved (type of aggression: Cohen's *k* = 1.0; number of calls: ICC = 1.0). For the duration of the encounter and the total number of bystander birds present, almost perfect agreement was achieved (duration: ICC = 0.999; number of bystanders: ICC = 0.997; all *n* = 16).

All bystander birds were categorized as kin or non-kin of the aggressor and the victim, respectively, based on their pairwise relatedness coefficient (see electronic supplementary material). Only individuals with an *r*-value greater than 0.368 (full siblings/parent-offspring) were labelled ‘kin'.

Additionally, based on focal observations on dyadic affiliative interactions of sitting in close contact (within one body's length) and allopreening (preening the plumage of a partner), bonding partners of the aggressor and the victim among the bystanders were identified. Focal observations lasted 1–5 min, and a total of 1580 min of 50 individuals (mean ± s.e.: 2.24 ± 0.07 min per focal observation) was used. Territorial breeding pairs were categorized as pair bonded (PB). Non-breeder individuals that were repeatedly observed engaging in reciprocal affiliative behaviours with one single partner were categorized as strongly bonded (SB), non-breeders with several bonding partners or unidirectional affiliative interactions as loosely bonded (LB). Cases involving individuals that were never observed exchanging affiliative behaviours (non-bonded birds) were not included in the analysis as the possibility of their bonding partner(s) being present was not given. Bystanders having a pair, strong or loose bonding status to the aggressor or the victim were labelled ‘bonding partner'.

### (b) Relationship value

Social bonds can be characterized by the direct value for the partners as well as the level of compatibility and security over time [54]. To calculate a proxy for relationship value in ravens [55], the duration of allopreening the focal individual received from others and provided to others was corrected for the total observation time. The time an individual spent preening other birds was then subtracted from the time an individual had been preened by others to obtain the net benefit the subject gained from preening interactions. Individuals with negative outcomes were classified as having a low relationship value as they invested more in preening than they received. On the contrary, individuals with a positive outcome received more than they invested, and thus were classified as having relationships of high value.

### (c) Dominance hierarchy

*Ad libitum* observations [56] of dyadic agonistic interactions resulted in a total of 594 interactions involving 72 individuals. From this, dominance rank was determined by calculating asymmetric dyadic interaction rates in SOCPROG 2.6 for Matlab v. 8.5.0, release 2015a [57]. Dominance hierarchy was calculated taking the sex and age class of the individuals into account. The modified linearity index was *h*′ = 0.077[58] with a steepness of −0.066 when using proportions of wins. Modified David's scores [59] of each individual were extracted and normalized in order to produce scores ranging from 0 to 1; 0 being the lowest-ranking individual, and 1 the highest-ranking bird. From this, rank difference of opponents was calculated by subtracting the rank of the victim from the rank of the aggressor.

### (d) Statistics

All statistics were conducted in R v. 3.3.3 [60] for Mac OS X. In ravens [41] and other corvids [33], dominance rank is linked to sex, age class and bonding status. We first examined the factors sex, age class, bonding status, rank difference of victims and aggressors and the total number of birds present for possible multi-collinearity by calculating variance inflation factors using the HighstatLib v. 6 package [61]. We encountered multi-collinearity of rank difference with sex, age class and bonding status. To avoid collinear factors in the model [62], and overfitting due to too many fixed effects, the effects of age class, sex, bonding status and the birds in close proximity on the call rates were analysed separately using non-parametric Mann–Whitney *U*-tests. Pairwise comparisons were calculated using Mann–Whitney *U*-tests on sample sizes corrected for repeated measures of victims and aggressors. To control for the false discovery rate, Benjamini & Hochberg adjustment of *p*-values was applied [63]. The model investigating the effects of audience composition on call rates included the four binomial factors absence/presence of kin of victims, absence/presence of kin of aggressors, absence/presence of bonding partners of victims, absence/presence of bonding partners of the aggressors, rank difference of opponents and the total number of birds present. We further included all interactions between the presence/absence of kin and bonding partners of victims and aggressors to test the effects of the simultaneous absence/presence of kin and bonding partners of aggressors and victims. A generalized linear mixed odel (GLMM) was calculated with a negative binomial distribution and a log link function to account for overdispersion using the glmmADMB package v. 0.8.3.3 [64,65]. The identities of the opponents were entered as a random factor to account for repeated sampling. A step-wise elimination method was applied to determine the best fitting model based on lowest AIC values. Starting with the full model, predictors that lead to the largest reduction of AIC were dropped step-wise. The final model was found when no more predictors were left to remove that could have lowered AIC. Likelihood ratio tests were used to ensure that the removal of predictors improved the model fit. The coefficients of the null, full and final models are presented in table 1. Post hoc Mann–Whitney *U*-tests were calculated on estimated mean values derived from the final model.

**Table 1.** Outcomes of null, full and final GLMMs testing the effect of audience composition on victims' defensive call rates. Coefficients with estimated means (EM), standard errors (s.e.), effect sizes (*z*-value) and significances (*p*-value) are shown. Note: ‘absence’ of kin and bonding partners of victims and aggressors were set as reference points. Colons denote interactions between coefficients.

model	coefficients	EM	s.e.	*z*-value	*p*-value
null model (AIC = 552.3)	(intercept)	1.741	0.08	22.48	<0.0001
full model (AIC = 495.2)	(intercept)	1.703	0.34	4.99	<0.0001
	kin of aggressors	−0.131	0.35	−0.37	0.7093
	kin of victims	0.588	0.30	1.93	0.0536
	bonding partner of aggressors	−0.574	0.32	−1.82	0.0686
	bonding partner of victims	0.109	0.46	0.24	0.8139
	rank difference of opponents	0.001	0.28	0.0	0.9998
	total number of birds present	−0.002	0.01	−0.20	0.8415
	kin of aggressors : kin of victims	0.185	0.34	0.55	0.5839
	kin of aggressors : bonding partner of aggressors	0.208	0.32	0.65	0.5157
	kin of aggressors : bonding partner of victims	−0.130	0.26	−0.50	0.6187
	kin of victims: bonding partner of aggressors	−0.171	0.34	−0.50	0.6136
	kin of victims : bonding partner of victims	−0.484	0.45	−1.07	0.2866
	bonding partner of aggressors : bonding partner of victims	0.181	0.45	0.40	0.6901
final model (AIC = 480.5)	(intercept)	1.698	0.16	10.79	<0.0001
	kin of victims	0.578	0.16	3.70	0.0002
	bonding partner of aggressors	−0.558	0.14	−3.93	<0.0001
	bonding partner of victims	0.200	0.20	1.00	0.3165
	kin of victims : bonding partner of victims	−0.634	0.25	−2.49	0.0129

## 3. Results

### (a) Audience composition

**Figure 1.**
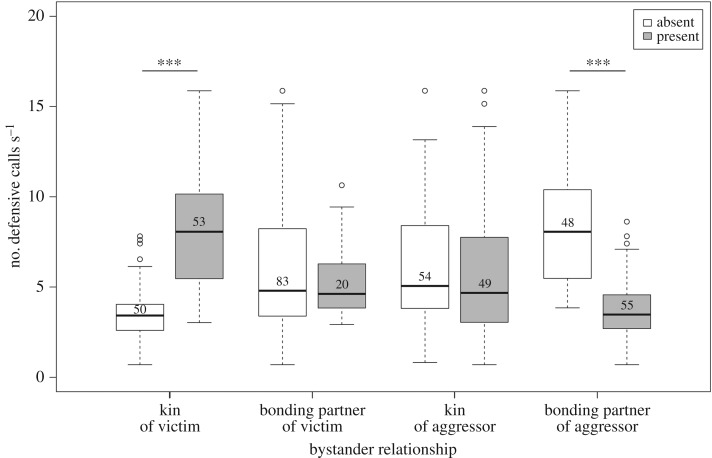
Defensive call rates of the victims with respect to the absence or presence of kin and bonding partners of the victims and the aggressors in the bystander audience. Values are estimates means derived from the GLMM. Boxes delineate interquartile ranges (IQR, 3rd–1st quartile), bold lines show the median, circles indicate outliers and whiskers represent minimum and maximum values excluding outliers (1.5 × IQR). Numbers within the boxes refer to sample sizes for each category. Asterisks denote significant differences in post hoc Mann–Whitney *U*-tests within the categories (***p < 0.001).

The final model investigating the influence of audience composition to victims' call rates contained as significant factors the presence/absence of bonding partners of the aggressors, the presence/absence of kin of victims and a significant interaction between the presence/absence of bonding partners of victims and kin of victims (table 1). Post hoc tests showed that victims' call rates were lower when bonding partners of the aggressor were present (Mann–Whitney *U*-test: *U* = 1878.0, *n*_1_ = 40, *n*_2_ = 50, *p* < 0.001; table 2). The opposite was found for the presence/absence of kin of victims: victims called at higher rates when their kin were present when compared to when their kin were absent (*U* = 155.0, *n*_1_ = 48, *n*_2_ = 41, *p* < 0.001; figure 1). In the cases where victims' kin were present, the average number of victims' kin present was 1.38 ± 0.66 (s.d.), and had no significant influence on victims' call rates (Spearman *ρ*: *r_s_* = −0.22, *p* = 0.1742).

**Table 2.** Post hoc Mann–Whitney *U*-tests of variations in mean call rates in the presence/absence of victims' and aggressors’ kin and bonding partners (*n_1,2_* indicates sample sizes). Original *p*-values and values adjusted after Benjamini & Hochberg (*p*_FDR_) are shown. Italicized values indicate significant differences after controlling for the false discovery rate.

pairwise comparisons	*n*_1,2_	*U*	*p*	*p*_FDR_
kin of victim: present–absent	41,48	155.0	<0.001	*<0.001*
kin of aggressor: present–absent	44,45	1167.5	0.146	0.195
partner of victim present–absent	20,67	676.5	0.952	0.952
partner of aggressor present–absent	50,40	1878.0	<0.001	*<0.001*

**Figure 2.**
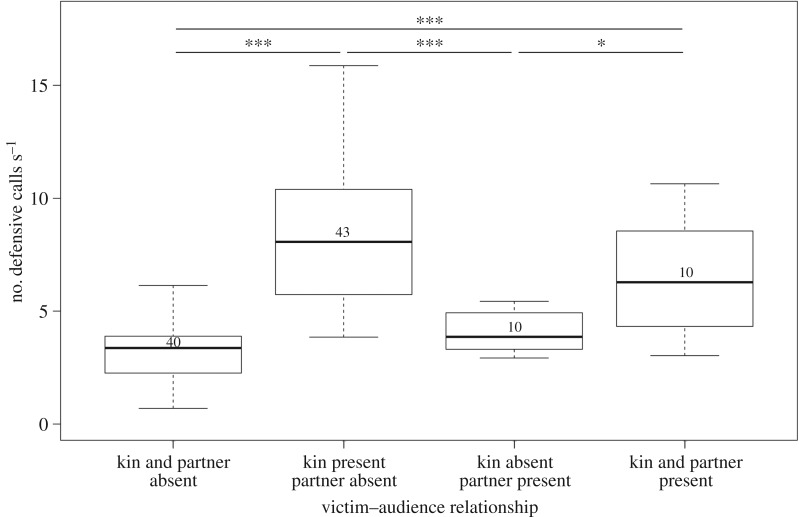
Victims' estimated mean call rates with respect to the simultaneous absence/presence of victims' kin and bonding partners. Numbers inside the boxes denote the number of cases per category. Boxes delineate IQR, bold lines represent the median and whiskers represent minimum and maximum values excluding outliers (1.5 × IQR), which are not shown. Asterisks indicate significant differences in post hoc Mann–Whitney *U*-tests (*p < 0.05, ***p < 0.001).

The interaction effect showed that call rates were high when both kin and bonding partners of victims were present at the same time (figure 2). Post hoc analysis revealed that victims' call rates were significantly higher when both kin and bonding partners of victims were present when compared to when both were absent (Mann–Whitney *U*-test: *U* = 52.0, *n*_1_ = 10, *n*_2_ = 38, *p* < 0.001; table 3), and when only kin of victims were present when compared to when both kin and partners were absent (*U* = 71.0, *n*_1_ = 34, *n*_2_ = 38, *p* < 0.001). Furthermore, call rates were significantly higher when only victims' kin were present when compared with when only victims' bonding partners were present (*U* = 315.0, *n*_1_ = 34, *n*_2_ = 10, *p* < 0.001), and when both kin and bonding partners were present (*U* = 237.0, *n*_1_ = 10, *n*_2_ = 10, *p* < 0.039). Call rates did not vary between other constellations of the presence/absence of victims' kin and bonding partners, indicating that the presence of victims' bonding partners alone did not result in a significant increase in call rates, but only in the simultaneous presence with victims' kin.

**Table 3.** Post hoc Mann–Whitney *U*-tests of variations in mean call rates in the presence/absence of victims' kin and victims’ bonding partners (*n*_1,2_ indicates sample sizes). Original *p*-values and values adjusted after Benjamini & Hochberg (*p*_FDR_) are shown. Italicized values indicate significant differences after controlling for the false discovery rate.

pairwise comparisons	*n*_1,2_	*U*	*p*	*p*_FDR_
both absent–only kin present	38,34	71.0	<0.001	*<0.001*
both absent–only partner present	38,10	116.5	0.064	0.064
both absent–both present	38,10	52.0	<0.001	*<0.001*
only kin present–only partner present	34,10	315.0	<0.001	*<0.001*
only kin present–both present	34,10	237.0	0.062	0.064
only partner present–both present	10,10	80.0	0.026	*0.039*

If we consider call rates in the absence of important social allies (kin of victims and bonding partners of aggressors) as a baseline, we find that call rates were significantly higher in this baseline (median ± s.e. = 5.11 ± 0.78) than when kin of victims were absent and bonding partners of aggressors were present (median ± s.e: 3.37 ± 0.24; *U* = 21.0, *n*_1_ = 4, *n*_2_ = 44, *p* = 0.013). On the contrary, call rates in baseline were significantly lower than when kin of victims were present and bonding partners of aggressors were absent (median ± s.e.: 9.27 ± 0.89; *U* = 122.0, *n*_1_ = 4, *n*_2_ = 36, *p* = 0.025).

### (b) Relationship value

Focal observations on affiliative behaviours outside the feeding context revealed that victims provided more preening to their bonding partners than they received (*n*_high_ = 10, *n*_low_ = 19); the opposite was true for aggressors, who received more preening from their partners than they provided (*n*_high_ = 15, *n*_low_ = 13). These differences in relative preening investment indicate that the victims' relationships to their bonding partners were of different quality than those of aggressors: as victims had to invest much in preening, the value of their relationships was probably low; aggressors, on the contrary, were the focus of preening and thus their relationships with their partner were probably of high value for them.

### (c) Other factors influencing call rates

Victims' call rates were not affected by the close proximity of potential social allies (bonding partners and kin) of victims and aggressors at the feeding site (table 4a). Age class, sex and bonding status of aggressors and victims did not influence victims' call rates (table 4*b*–*d*).

**Table 4.** Pairwise Mann–Whitney *U*-tests for defensive call rates and (*a*) the close proximity of kin and bonding partners to aggressors and victims, (*b*) sex, (*c*) age class and (*d*) bonding status of aggressors and victims. *n*_1,2_ denotes the number of cases per category. Original *p*-values and values adjusted after Benjamini & Hochberg (*p*_FDR_) are shown.

pairwise comparisons	*n*_1,2_	*U*	*P*	*p*_FDR_
(*a*) *close proximity*				
kin of aggressors (close/not close)	4,26	63.0	0.536	0.674
bonding partners of aggressors (close/not close)	8,25	130.0	0.220	0.440
kin of victims (close/not close)	6,27	37.0	0.040	0.240
bonding partners of victims (close/not close)	1,29	8.0	0.600	0.674
*(b) sex*				
aggressors (male/female)	17,11	74.0	0.378	0.648
victims (male/female)	14,15	159.0	0.018	0.216
*(c) age class*				
aggressors (adult/subadult)	15,13	66.0	0.156	0.396
victims (adult/subadult)	11,18	106.0	0.774	0.774
*(d) bonding status*				
aggressors (PB/SB)	2,11	15.0	0.489	0.674
aggressors (PB/LB)	2,15	19.0	0.618	0.674
aggressors (SB/LB)	11,15	117.0	0.077	0.308
victims (SB/LB)	7,22	49.0	0.165	0.396

## 4. Discussion

Our results show that victims modulate their call rates according to the presence of particular individuals in the audience. Compared with audience effects found in avian alarm calls [19] or food calls [20], signalling ravens not only take into account bystanders with whom they have a valuable relationship, but also bystanders that have a valuable relationship to their aggressors.

The differences in the effect of bonding partners and kin probably indicate differences in relationship quality for victims and aggressors. The presence of kin seemed to have a stronger effect on victims' call rates than the presence of bonding partners. At the same time, the quality of victims' bonds to non-kin partners was found to be weaker than those of aggressors and their bonding partners. These findings suggest that victims are able to assess the strength of their social bonds, and focus on their kin in the lack of valuable social bonds with non-kin bonding partners. Similar effects of kinship are known from studies in captive ravens: siblings tend to have valuable relationships [49], support each other during conflicts [42] and provide post-conflict affiliation [43]. However, kin effects have not been reported from free-ranging ravens so far, possibly because levels of relatedness in raven foraging groups are low [39] (except for young ravens, which mainly associate with their siblings [66]). Note that this pattern also holds in our study population: in cases where victims' kin was present, the number of kin was on average 1.38.

Remarkably, victims also seem to have knowledge of the social bonds of their aggressors, and possibly use this knowledge during conflicts to anticipate third-party support to the aggressor. The selective suppression of calling suggests that victims control their vocalization so as to avoid the attention of allies of their aggressors, which reflects triadic awareness. This interpretation is supported by experimental studies on ravens' ability to form representations of third-party relationships [35]. Our results are also in line with observations on triadic intervention patterns during affiliative interactions in free-ranging ravens [67], as well as on reciprocity in social support during agonistic interactions among alliance partners in captive ravens [42]. Although the current findings corroborate that free-ranging ravens recognize own *and* others' social bonds [67], they hint towards difficulties in recognizing others' kin relationships, as victims did not decrease call rates when kin of aggressors were present. These results are in contrast to those of most primates, which seem to recognize different types of relationships of others, including rank, simultaneously. For instance, free-ranging baboons (*Papio cynocephalus ursinus*) responded more strongly to calls of their own and the opponents' kin in simulated agonistic interactions in a playback experiment [68], and white-faced capuchins (*Cebus capucinus*) were more likely to solicit help from individuals with whom they had stronger affiliative relationships, and also from individuals that were higher in rank than their opponents [69]. Unlike most primates, ravens do not live in stable groups structured by kin such as matrilines. Instead, ravens' social organization is characterized by high fission–fusion dynamics [38], which might provide only limited opportunities to learn of others' kin relations.

If the presence of any audience, irrespective of its composition, would have caused changes in victims' call rates, the expected pattern would have been a general increase or decrease in call rates with an increase in the number of bystanders in the audience, as a big audience may either facilitate or inhibit calling in victims. Yet the total number of birds did not influence victims' call rates. One could argue that aggressors, in the presence of their bonding partners, are primed to be more aggressive due to the potential support they could receive, but the agonistic interactions in focus were conflicts of moderate intensity (forced retreats), and the degree of aggression from the attacking birds were always the same. Thus, a possible priming effect in aggressors is controlled for when investigating differential responses of victims. Furthermore, social allies were rarely found to be in the direct vicinity (within 1 m) of the opponents, and their proximity did not affect victims' call rates. Thus, we can rule out that the bystanders directly influenced the behaviour of the victims (e.g. by sitting close). This suggests that changes in victims’ call rates did not vary in response to bystander behaviour, but did vary in response to victims' knowledge of social relationships. Likewise, the total number of birds present did not influence victims' call rates. This finding speaks against the possibility that victims adjusted their calling to group size in general.

How ravens have acquired their skills remains an open question to be investigated in future studies. It might be that they have learned to selectively suppress or increase calling in response to audience composition. Indeed, the daily competition for food offers plenty of opportunities for repeated interactions, but the high dynamics in group composition, with individuals regularly coming and going, would require ravens to show a high flexibility in using the learned information according to context. Another possibility would be that the audience effect rests on an affective, arousal-based diminishing of calling in the context of aggressive individuals and/or in social groups of strangers (without any kin/familiar birds present). Investigating the role of emotions in respect to the suppression of calling is certainly a promising next step; however, the specificity of call suppression observed in this study speaks against an interpretation solely based on affective states. In addition, we suggest playback experiments to test the responses of the audience to varying defensive call rates (i.e. by manipulating the calling frequencies depending on the audience composition).

In summary, we found that ravens flexibly adjusted their signalling behaviour to audience composition during agonistic interactions, taking triadic affiliative social relationships into account. The results add to the emerging picture of sophisticated social knowledge in ravens and support the idea of social life being a driving force for socio-cognitive skills in corvids [70].

## Supplementary Material

Sexing and kinship analysis

## Data accessibility

Electronic supplementary materials are available for this paper. Data available from the Dryad Digital Repository: http://dx.doi.org/10.5061/dryad.64q763h [71].
