# Supplementary material for: Attacked ravens flexibly adjust signalling behaviour according to audience composition
Source: Proc Biol Sci. 2018 Jun 6;285(1880):20180375. doi: 10.1098/rspb.2018.0375 (PMC6015854; doi:10.1098/rspb.2018.0375)
Supplement: Sexing and kinship analysis [file rspb20180375supp1.pdf]

Supplementary Materials referred to in

“Attacked ravens flexibly adjust signalling behaviour according to audience composition” by Georgine Szipl, Eva Ringler & Thomas Bugnyar,

published in Proceedings B (DOI: 10.1098/rspb.2018.0375)

Sexing and kinship analysis

Blood samples of 176 ravens were collected during the marking procedure. Genomic DNA was isolated using a Proteinase K digestion followed by a standard phenol–chloroform protocol [1]. For the sex determination PCR protocol [2] was performed PCR using the primers P8 (5’-CTCCCAAGGATGAGRAAYTG-3’) and P2 (5’-TCTGCATCGCTAAATCCTTT-3’). The amplified products were separated on 3% agarose gels stained with ethidium bromide to distinguish between males (only a single Z-band) and females (Z- and W-bands). In order to obtain individual genotypes for relatedness analyses all individuals were genotyped at 15 microsatellite loci. PCR amplifications were performed using reaction volumes of 10 μL containing about 20-50 ng of genomic DNA, 0.2 mm of each dNTP, 1 μm of each forward and reverse primer, 0.5 U of Taq DNA polymerase (Axon) and 1 μL of 10× NH_4 reaction buffer (Axon), at a final concentration of 1.5 mm $MgCl_2$. The following PCR programme was used: 8 min at 95 °C, 39 cycles at 95 °C for 45 s, the primer specific annealing temperature for 45 s, 72 °C for 45 s, followed by a final extension step for 8 min at 72 °C. Differences in the sizes of the amplified alleles and in the fluorescent dye labels of the primers allowed for pooling of multiple loci for the subsequent sequencing process. The pooled products were then diluted with water 1:30, mixed with HiDiformamid and the internal size standard ROX500 (Applied Biosystems), and run on an ABI 3130xl Genetic Analyser. Alleles were manually inspected using Peakscanner Software (Applied Biosystems), and final allele sizes were determined using TANDEM v1.08 [3]. The program KINGROUP v2 [4] was used to determine pairwise relatedness coefficients ‘*r*’ [5] for all

possible dyads. We used the implemented simulation function to obtain reference intervals (first to third quartile) for expected pairwise relatedness values for 100 full siblings, 100 half siblings, and 100 unrelated individuals based on the actual allele frequencies of our focal population. Through this approach we obtained the most probable reference intervals for first- and second-order relatives, as well as for unrelated individuals, which were [0.365;0.573],[0.149;0.370], and [-0.120;0.122], respectively. Accordingly, we defined full-siblings as all individual pairs with *r*-values > 0.368, half siblings with values between 0.135 and 0.368, and unrelated individuals with values below 0.135.

Supplementary References

1. Sambrook, J., Fritsch, E. F. & Maniatis, T. 1989 *Molecular cloning: A laboratory manual*. 2nd edn. New York: Cold Spring Harbor Laboratory Press.

2. Griffiths, R., Double, M. C., Orr, K. & Dawson, R. J. G. 1998 A DNA test to sex most birds. *Mol Ecol* **7**, 1071–1075.

3. Matschiner, M. & Salzburger, W. 2009 TANDEM: integrating automated allele binning into genetics and genomics workflows. *Bioinformatics* **25**, 1982–1983. (doi:10.1093/bioinformatics/btp303)

4. Konovalov, D. A., Manning, C. & Henshaw, M. T. 2004 KINGROUP: a program for pedigree relationship reconstruction and kin group assignments using genetic markers. *Mol Ecol Notes* **4**, 779–782. (doi:10.1111/j.1471-8286.2004.00796.x)

5. Queller, D. C. & Goodnight, K. F. 1989 Estimating Relatedness Using Genetic Markers. *Evolution* **43**, 258–275.
